# A mosaic *PIK3CA* variant in a young adult with diffuse gastric cancer: case report

**DOI:** 10.1038/s41431-021-00853-6

**Published:** 2021-06-01

**Authors:** Iris B. A. W. te Paske, José Garcia-Pelaez, Anna K. Sommer, Leslie Matalonga, Teresa Starzynska, Anna Jakubowska, Solve-RD-GENTURIS group,, Laura Valle, Gabriel Capella, Stefan Aretz, Elke Holinski-Feder, Verena Steinke-Lange, Andreas Laner, Evelin Schröck, Andreas Rump, Marjolijn Ligtenberg, Alexander Hoischen, Nicoline Geverink, D. Gareth Evans, Marc Tischkowitz, Steven Laurie, Rachel S. van der Post, Jan Lubinski, Carla Oliveira, Nicoline Hoogerbrugge, Richarda M. de Voer

**Affiliations:** 1 Department of Human Genetics, Radboud University Medical Center, Radboud Institute for Molecular Life Sciences, Nijmegen, the Netherlands; 2 i3S, Institute for Research and Innovation in Health of the University of Porto, Porto, Portugal; 3 Ipatimup, Institute of Molecular Pathology and Immunology of the University of Porto, Porto, Portugal; 4 FMUP, Faculty of Medicine of the University of Porto, Porto, Portugal; 5 Institute of Human Genetics, University of Bonn, Bonn, Germany; 6 CNAG-CRG, Centre for Genomic Regulation, Barcelona Institute of Science and Technology, Barcelona, Spain; 7 Department of Gastroenterology, Pomeranian Medical University, Szczecin, Poland; 8 Department of Genetics and Pathology, Pomeranian Medical University, Szczecin, Poland; 9 Independent Laboratory of Molecular Biology and Genetic Diagnostics, Pomeranian Medical University, Szczecin, Poland; 10 Department of Pathology, Radboud University Medical Center, Radboud Institute for Molecular Life Sciences, Nijmegen, the Netherlands; 11 Catalan Institute of Oncology, IDIBELL, Barcelona, Spain; 12 MGZ-Center of Medical Genetics, Munich, Germany; 13 Institute for Clinical Genetics, Technical University Dresden, Dresden, Germany; 14 Department of Pathology, Radboud University Medical Center, Nijmegen, the Netherlands; 15 Department of Human Genetics, Radboud University Medical Center, Radboud Institute for Molecular Life Sciences, Nijmegen, the Netherlands; 16 Department of Urology, Radboud University Medical Center, Nijmegen, the Netherlands; 17 Manchester Centre for Genomic Medicine, Manchester, UK; 18 Department of Medical Genetics, University of Cambridge, Cambridge, UK; 19 CNAG-CRG, Centre for Genomic Regulation, Barcelona Institute of Science and Technology, Barcelona, Spain

**Keywords:** Cancer genomics, Gastric cancer

## Abstract

Hereditary diffuse gastric cancer (HDGC) is associated with germline deleterious variants in *CDH1* and *CTNNA1*. The majority of HDGC-suspected patients are still genetically unresolved, raising the need for identification of novel HDGC predisposing genes. Under the collaborative environment of the SOLVE-RD consortium, re-analysis of whole-exome sequencing data from unresolved gastric cancer cases (*n* = 83) identified a mosaic missense variant in *PIK3CA* in a 25-year-old female with diffuse gastric cancer (DGC) without familial history for cancer. The variant, c.3140A>G p.(His1047Arg), a known cancer-related somatic hotspot, was present at a low variant allele frequency (18%) in leukocyte-derived DNA. Somatic variants in *PIK3CA* are usually associated with overgrowth, a phenotype that was not observed in this patient. This report highlights mosaicism as a potential, and understudied, mechanism in the etiology of DGC.

## Introduction

In ~10% of the gastric cancer (GC) cases familial aggregation is observed [1]. Two monogenic GC-associated syndromes have been described so far: (i) Hereditary Diffuse Gastric Cancer syndrome (HDGC; MIM 137215) [2], and (ii) Gastric Adenocarcinoma and Proximal Polyposis of the Stomach syndrome (GAPPS) [3].

HDGC is associated with germline deleterious variants in *CDH1* and *CTNNA1*. However, deleterious variants in *CDH1* or *CTNNA1* are identified in only 10-40% of families fulfilling HDGC clinical criteria [2, 4, 5]. *PALB2* and *MYD88* are considered candidate genes for HDGC that need further confirmation [6, 7].

Currently, a large proportion of clinically and pathologically confirmed HDGC families and individuals developing diffuse gastric cancer (DGC) at very young age remain genetically unresolved, raising the need for research on novel inherited predisposing factors. Here, we report the case of a 25-year-old female with DGC, in whom a mosaic *PIK3CA* variant was identified in leukocyte-derived DNA by re-analysis of whole-exome sequencing (WES) data by the SOLVE-RD consortium.

## Materials and methods

### Patient cohort

Between January 2019 and September 2019 members of the European Reference Network on genetic tumor risk syndromes (ERN-GENTURIS) submitted 294 unresolved germline WES and whole-genome sequencing (WGS) datasets from genetic tumor risk syndrome suspected individuals for re-analysis to SOLVE-RD. From this initial 294 unresolved cases, 83 had a phenotype of non-GAPPS gastric cancer that were reanalyzed in the present study. This study was approved by local ethics committees (local study identification numbers: Ipatimup: 152/18; University of Cambridge: 97/5/032; Radboudumc: 2018-4985, 2018-4986).

### Re-analysis of WES data

For each case, sequencing reads were processed using the RD-Connect Genome-Phenome Analysis Platform standardized analysis pipeline based upon GATK3.6 best practices (further information: http://solve-rd.eu/ and http://rd-connect.eu/). Subsequently, all variants with a read-depth ≥8-fold and a genotype quality of ≥20 were selected for downstream processing and interpretation. Each variant was annotated using VEP from Ensembl as described by Matalonga et al. [8]. For re-analysis of WES data submitted by ERN-GENTURIS, variants present in a gene list composed of 229 genes associated with genetic tumor risk syndromes (Supplementary Table 1) were assessed for their pathogenicity as annotated by ClinVar. Variants predicted to be pathogenic or likely pathogenic were followed up for interpretation and validation.

Raw whole-exome data of this patient is available via the European Genome-Phenome Archive (EGA; accession number EGAZ00001545545). The variant described in this manuscript is submitted to the Leiden Open Variant Database (LOVD; https://databases.lovd.nl/shared, Individual ID number 00327392).

### Validation by smMIP sequencing

Genomic DNA extracted from peripheral blood was screened in triplicate for hotspot regions (codons 345, 420, 539-554, 1043-1050) of *PIK3CA* (NM_006218.4) using small molecule molecular inversion probes (smMIP) sequencing as described by Steeghs et al. [9].

## Results

### Clinical phenotype

A 25-year-old female presented with abdominal discomfort for two months when endoscopic investigation of the stomach revealed a gastric mass. After total gastrectomy a diffuse type gastric adenocarcinoma (T2N1M0) was identified in the antrum of the stomach. The patient tested negative for *H. pylori* infection and no other coexisting disorders or congenital abnormalities were reported. Immunohistochemical P53 expression in the tumor showed a wild-type pattern. The patient was treated with adjuvant 5-fluorouracil, etoposide and cisplatin, but she deceased 12 months after diagnosis due to peritoneal metastasis, ileus of the small bowel, ascites and cachexia.

### Germline analysis

**Fig. 1:**
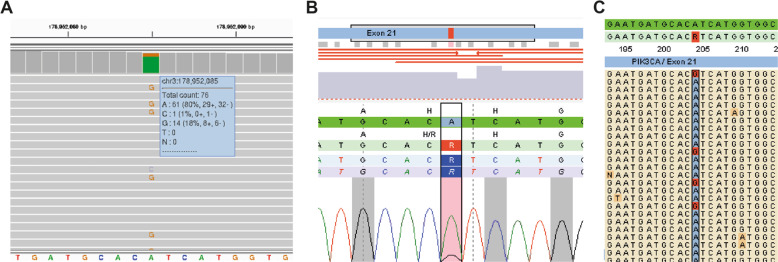
Mosaic *PIK3CA* c.3140A>G variant found in leukocyte DNA of the proband. **A** Screenshot of the Integrative Genomics Viewer. Alternative alleles at *PIK3CA* c.3140 position are marked. Variant details are shown in the panel on the right. **B** Screenshot of one outcome of the in triplicate smMIP screened leukocyte-derived DNA from the proband. The *PIK3CA* (c.3140A>G; p.(His1047Arg)) variant is marked in red. **C** Screenshot of individual reads in smMIP analysis. Alternative alleles at *PIK3CA* c.3140 position are marked in red.

The patient did not have a family history of gastric cancer, but due to her very early-onset of DGC she met the clinical criteria for testing for HDGC [10]. In absence of a germline *CDH1* variant that is predicted to impair protein function, the patient was selected for WES analysis, but putative disruptive variants in cancer predisposition genes were not identified [11]. Re-analysis of the generated WES data within the SOLVE-RD consortium revealed one ClinVar-reported missense variant in *PIK3CA* (NM_006218.4; c.3140A>G; p.(His1047Arg)), a gene associated with somatic overgrowth syndromes, but not previously associated with gastric cancer [12, 13]. This variant was present in blood leukocytes at a variant allele frequency (VAF) of 18% (14/76 sequencing reads), suggesting presence in mosaic state (Fig. 1A). Triplicate smMIP sequencing confirmed mosaicism of *PIK3CA* c.3140A>G, with a VAF ranging from 13% to 16% (Fig. 1B,C). Germline genetic testing was performed on genomic DNA extracted from leukocytes that were taken from the patient prior to chemotherapy. No tumor tissue was available for further histopathological assessment or somatic sequencing.

### Variant characteristics

*PIK3CA* c.3140 is a known somatic hotspot location in many cancer types, including gastric tumors [14]. Variants in *PIK3CA* are found in 15% of the stomach adenocarcinomas (5 studies; 739 samples) and 18% of these cancers harbor the p.(His1047Arg) missense variant [15, 16]. We investigated the frequency of *PIK3CA* c.3140A>G p.(His1047Arg) in population datasets not enriched for tumor associated phenotypes. The variant was not found in >13,000 individuals with WES data (inhouse database) or in >2,000 individuals from other (non-cancer) SOLVE-RD cohorts. *PIK3CA* c.3140A>G was only detected in 1 out of >120,000 gnomAD individuals. While little details are available, the variant was identified in a female non-Finnish European aged 35–40 years, at a VAF of 25–30%, also suggestive of somatic mosaicism [17]. Interestingly, the germline WES data from this female originated from The Cancer Genome Atlas, indicating that this individual too developed cancer at a young age (≤40 years).

## Discussion

In this study, we present the case of a woman with early-onset DGC in whom neither targeted *CDH1* and *CTNNA1* variant screening, nor a previous WES analysis provided a genetic diagnosis. Re-analysis of the WES data revealed a mosaic *PIK3CA* c.3140A>G p.(His1047Arg) variant, suggestive of a potential role in the patient’s phenotype and early-onset of diffuse type gastric cancer.

Mosaic *PIK3CA* variants have been described in *PIK3CA*-related overgrowth syndromes (PROS), syndromes marked by congenital or early-onset of sporadic segmental tissue overgrowth, vascular malformations and mosaic skin lesions [18]. A constitutional heterozygous *Pik3ca*^H1047R^ murine model is embryonically lethal [19], an observation that is consistent with the mosaic status of *PIK3CA* in PROS and in gnomAD. For *PIK3CA* somatic mosaicism, the timing and location when a *PIK3CA* is variant introduced during postzygotic development likely determines the phenotypic presentation of malignancies [13]. For PROS it is reported that mosaic *PIK3CA* variants arise in ectodermal and mesodermal tissues, whereas the digestive tract, including the stomach, arises from endoderm. Since no clear signs of overgrowth or dysmorphologies were observed, but the variant is detected in leukocyte-derived DNA, the variant may be present in the endoderm and mesoderm layer only. The mosaic state of the variant could be the result of a postzygotic introduction of the variant in the mesendoderm layer [20]. Blood used in the genetic analyses in this study was obtained before chemotherapy, which rules out a scenario of clonal expansion of the variant due to the selective pressure of chemotherapeutic agents.

The initial WES analysis, described by Vogelaar et al., was directed towards the identification of rare high-confidence nonsynonymous germline variants (≥20% variant reads) in known hereditary cancer genes or genes involved in GC development. This approach reduces the chance of identification of mosaicism, as these variants often do not meet the quality threshold of ≥20% variant reads [11]. By decreasing the VAF cut-off, in combination with ClinVar assessment, mosaic disease-causing variants associated with impaired protein function can be identified, a strategy that is not widely applied to WES and WGS studies. However, WES/WGS data alone is not sufficient to confirm etiology, and further evidence for pathogenicity, using a multifactorial approach and other tissue sources is critical for this. Unfortunately, due to the historical age of the case, no additional (tumor) tissue samples could be obtained from the patient to demonstrate the presence of the variant in tissues derived from different embryonic layers and the neoplastic cells of the gastric tumor.

To our knowledge, there is no literature describing mosaic *PIK3CA* variants in association with DGC. Within the field of unresolved rare disorders, the identification of causative variants and molecular diagnosis is challenging, as an obvious the genotype-phenotype often cannot be found. However, a similar case of early-onset cancer in combination with a mosaic *PIK3CA* variant is reported in the gnomAD-TCGA database, supporting the increased cancer risk associated with mosaicism for this variant. Our finding suggests that mosaic predisposing variants are potentially understudied in individuals suspected of having a genetic tumor risk syndrome. As such, it will be of interest to investigate the prevalence of mosaic *PIK3CA* variants in DGC and other cancers diagnosed in young adults. Detection of mosaicism has implications for relatives as well. As heterozygosity for *PIK3CA* c.3140A>G is considered lethal, relatives do most probably not have an increased risk for cancer.

To conclude, further studies are needed to confirm the potential role of *PIK3CA* mosaicism in cancer susceptibility. However, this report demonstrates the success of an improved approach for (mosaic) variant discovery. The reported diagnostic value of exome re-analysis should stimulate the field to re-analyze exome data of genetically undiagnosed genetic tumor risk syndrome patients by similar approaches. Furthermore, this report underlines the complexity that rare disease patients may face awaiting their genetic diagnosis. For many disease genes we may not yet know the full phenotypic presentation, which is especially challenging in genes like *PIK3CA*, where the presentation of the clinical phenotype is dependent on the timing of the postzygotic event.

## Supplementary information

Supplementary table 1
